# Reaction Time and Mortality from the Major Causes of Death: The NHANES-III Study

**DOI:** 10.1371/journal.pone.0082959

**Published:** 2014-01-29

**Authors:** Gareth Hagger-Johnson, Ian J. Deary, Carolyn A. Davies, Alexander Weiss, G. David Batty

**Affiliations:** 1 Department of Epidemiology and Public Health, University College London (UCL), London, United Kingdom; 2 Department of Psychology, Centre for Cognitive Ageing and Cognitive Epidemiology, University of Edinburgh, Edinburgh, United Kingdom; 3 Medical Research Council (MRC)/Chief Scientist Office (CSO) Social and Public Health Sciences Unit, University of Glasgow, Glasgow, United Kingdom; Innsbruck Medical University, Austria

## Abstract

**Objective:**

Studies examining the relation of information processing speed, as measured by reaction time, with mortality are scarce. We explored these associations in a representative sample of the US population.

**Methods:**

Participants were 5,134 adults (2,342 men) aged 20–59 years from the Third National Health and Nutrition Examination Survey (NHANES III, 1988–94).

**Results:**

Adjusted for age, sex, and ethnic minority status, a 1 SD slower reaction time was associated with a raised risk of mortality from all-causes (HR = 1.25, 95% CI 1.12, 1.39) and cardiovascular disease (CVD) (HR = 1.36, 95% CI 1.17, 1.58). Having 1 SD more variable reaction time was also associated with greater risk of all-cause (HR = 1.36, 95% CI 1.19, 1.55) and CVD (HR = 1.50, 95% CI 1.33, 1.70) mortality. No associations were observed for cancer mortality. The magnitude of the relationships was comparable in size to established risk factors in this dataset, such as smoking.

**Interpretation:**

Alongside better-established risk factors, reaction time is associated with increased risk of premature death and cardiovascular disease. It is a candidate risk factor for all-cause and cause-specific mortality.

## Introduction

Slower and more variable simple reaction times are associated with elevated rates of all-cause [1], [2], [3], [4] and cardiovascular disease (CVD) [2], [3], [5] mortality risk. Simple reaction time is thought to be a more basic index of neuropsychological functioning than choice reaction time. Choice reaction time involves choosing one of several response options, which is more cognitively complex. Reaction time variability represents variability across multiple trials within each participant's performance during a testing session. Such variability is also thought to be an important index of neuropsychological functioning [6]. Reliability of reaction time as a measure is increased by averaging scores over numerous trials.

As a measure of processing speed, reaction time is moderately inversely correlated with higher-level cognitive ability as assessed by psychometric tests: people with higher cognitive ability tend to have shorter, less variable reaction times [7]. Lower cognitive ability measured in childhood [8], [9], [10], [11], [12], [13], [14], [15], [16], [17], [18], [19], early adulthood [20], [21], [22], [23], [24], [25], and old age [4], [5], [26] is also associated with greater risk of all-cause [4], [13], [18], [23], [25], [26], [27], [28] and cardiovascular disease [5], [24], [27], [29] mortality. In a meta-analysis comprising 16 studies of over one million participants, a 1 standard deviation increase in cognitive ability in childhood was associated with 24% lower risk of mortality [28]. Reaction time and cognitive ability may both predict mortality risk because they both measure important aspects of neuropsychological functioning or reflect the integrity of one or more bodily systems. However, reaction time is also seen to explain the IQ-mortality association [2] suggesting that it may mediate the association between more complex cognitive processes and mortality.

In most studies of mortality risk factors, cognition has been ascertained using standard, psychometric tests of intelligence which some commentators claim are not equally valid for adults from different cultural backgrounds. Compared to psychometric tests of intelligence, [30], [31] simple reaction time can be regarded as a ‘culture-reduced’ measure of cognitive ability. It also relatively quick to measure at low cost [32]. In studies to date, slower and more variable simple reaction times have been associated with all-cause and CVD mortality risk [1], [2], [3], [33].

Our aim was to examine the relation between slower and more variable simple reaction times, with cause-specific mortality, in a representative sample of the US civilian community-dwelling population.

## Participants and Methods

### Participants

The sampling strategy for the Third National Health and Nutrition Examination Survey (NHANES III, 1988–94) [34], [35] involved a complex, multi-stage, stratified and clustered design. The sample was representative of the community-dwelling population of the US. Participants completed a home-based interview, questionnaire and visited a mobile examination centre. The analytic sample comprised 5,134 adults (2,342 men) aged 20 to 59 with data on reaction time and who were followed for mortality for 15 years (378 deaths). Mortality status was ascertained following a probabilistic match between NHANES-III and the National Death Index, using death certificates. Mortality was specified as the underlying cause listed on each death certificate. Follow-up time was censored at death or end of follow-up, whichever came first. The July 1997 data file was used for analyses, which is available in a publically accessible database (http://www.cdc.gov/nchs/nhanes/nh3data.htm#1a).

### Measures

#### Reaction time

Reaction time was measured as part of the computerized Neurobehavioral Evaluation System 2 (NES2) [36], [37]. Participants were asked to depress a button immediately upon seeing a ‘0’ displayed on a screen. Mean reaction time across 50 trials was used for analysis. There was a random inter-stimulus interval ranging from 2.5 to 5 seconds. There were no practice trials.

#### Covariates

Age in years, sex and ethnic minority status (Non-Hispanic white vs. Non-Hispanic black, Mexican-American or other) were recorded. Educational attainment was denoted as the highest grade or year of regular school that the participant completed (range 1 to 17). Occupational social class was based on the participant's longest-held occupation, ranked from lowest (e.g. equipment cleaners) to highest (e.g. executives, administrators, and managers). Poverty-income ratio is an index of relative poverty, where scores of 1 or below indicate being at or above relative poverty.

#### Health behaviors

Participants reported the number of cigarettes smoked per day. Alcoholic drinks were defined as a 12-oz serving of beer, a 4-oz glass of wine, or an ounce of liquor; the number consumed weekly was recorded. To estimate saturated fat intake, a 24-hour dietary recall method conducted by interviewers. Participants self-reported all food and drink consumed in the previous 24 hours, which was used to estimate saturated fat consumption according to the USDA database. Respondents were asked how frequently they performed specific leisure time physical activities in the past month. We then classified participants as being physically active (moderate activity 5 or more times per week or vigorous activity 3 times per week), inactive (no moderate or vigorous activities), or insufficiently active (falling between these two categories) [38].

#### Cardiovascular disease risk factors

During the clinical examination, systolic and diastolic blood pressure were measured up to six times according to a standard protocol using a mercury sphygmomanometer. Body Mass Index (BMI) was computed from weight and standing height squared, using measurements taken in the examination. For descriptive analyses, overweight was defined as BMI 25–29.99 and obesity as ≥30. Serum cholesterol was measured enzymatically; levels of C-reactive protein were ascertained using a Behring latex-enhanced CRP assay. CRP values ≥3 are considered potentially indicative of cardiovascular disease risk [39].

### Statistical analysis

Having determined that the proportionality assumption had not been violated, Cox regression with years of follow-up as the timescale was performed in Mplus version 6.2. Sample weights were used to obtain corrected standard errors, allowing for the survey design which involved over-sampling of subgroups considered to have particular public health relevance (e.g. ethnic minorities and older adults). All reaction time scores were standardized to z-scores (mean = 0; standard deviation = 1) where higher scores indicate slower or more variable (i.e. disadvantage) reaction times. For descriptive analyses, means (for continuous variable) and proportions (for categorical variables) were age-adjusted. Missing data on variables other than the exposure and vital status were replaced using multiple imputation [40] of 40 datasets, corresponding to approximately 1 dataset per 1% missing data [41].

### Percent attenuation

To identify variables that might explain an association between reaction time and mortality, percentage attenuation following the addition of groups of confounders and possible mediators (hereafter, covariates) was calculated using the formula 100*[(B_Model 1_−B_Model 1+covariates_)/(B_Model1_)] where B is the logit (not the hazard ratio). Each group of variables (educational attainment, SES, health behaviours, CVD risk factors) was evaluated separately to reduce the likelihood of over-adjustment.

### Sensitivity analyses

Sensitivity analyses included comparing estimates following multiple imputation with estimates from models performed on participants with complete data, to identify possible sources of bias. We also repeated analysis after excluding participants who died within five years of neuropsychological assessment. This allowed us to evaluate the possible impact of reverse causality, that is, that participants may have worsening reaction time scores because they were already terminally ill. We also compared results in three age groups, to evaluate possible effect modification by age.

## Results

In preliminary analyses (not shown), we found no evidence that the reaction time-mortality associations differed by sex or ethnic minority status (p-values for interactions all >.05). We thus pooled data for men and women.

The baseline characteristics of the study population in relation to later vital status are shown in Table 1. A total of 378 (7.4%) participants died during 14.6 years of follow-up (104 cardiovascular deaths; 84 cancer deaths). Adjusted for age, participants who died were more likely to be male, have lower socio-economic position, were physically inactive, and smoked cigarettes and drank alcohol more heavily (Table 1).

**Table 1:** Baseline characteristics of the Analytic Sample According to Vital Status after 15 years of follow-up.

	Total	Alive	Dead	P-value
	(N = 5,134)	(N = 4756)	(N = 378)	
	N (valid %)	Age-adjusted % (95% CI)		
Male	2,342 (45.6)	44.7 (43.3, 46.1)	58.8 (52.8, 64.8)	<0.001
Ethnic minority	3,318 (64.6)	64.1 (62.8, 65.5)	65.1 (65.4, 76.1)	0.01
School grade 10 not completed	1,053 (20.6)	20.3 (19.1, 21.4)	23.5 (18.6, 28.3)	0.05
Low occupational class	1,564 (32.5)	30.4 (29.1, 31.7)	39.4 (33.4, 45.2)	<0.001
Current regular smoker	1,448 (35.7)	33.9 (32.3, 35.4)	54.9 (48.1, 61.7)	<0.001
>35 alcoholic drinks weekly	88 (2.1)	1.9 (1.5, 2.3)	4.4 (1.7, 7.1)	0.01
Physically inactive	939 (28.0)	27.5 (26.0, 29.1)	28.9 (22.7, 35.0)	0.09
Overweight or obese	3,000 (58.5)	58.6 (57.3, 60.0)	59.1 (53.0, 65.2)	0.47
C-reactive protein >3 mg/dL	44 (0.9)	0.8 (0.6, 1.1)	2.0 (0.1, 3.9)	0.18
	Mean (SD)	Age-adjusted mean (95% CI)		
Age in years at baseline	36.7 (11.0)	36.1 (35.8, 36.4)	44.3 (43.2, 45.4)	<0.001
Poverty/income ratio	2.45 (1.78)	2.50 (2.45, 2.55)	1.85 (1.66, 2.04)	<0.001
Saturated fat, g/day	28.9 (18.6)	28.9 (28.3, 29.4)	29.5 (27.5, 31.4)	0.55
Systolic blood pressure, mmHg (mean, SD)	118.6 (14.6)	118.2 (117.9, 118.6)	123.8 (122.4, 125.1)	<0.001
Serum cholesterol, mg/dL (mean, SD)	5.1 (1.1)	5.14 (5.11, 5.17)	5.05 (4.94, 5.15)	0.11
Simple reaction time, ms (mean, SD)	242.7 (58.0)	242.0 (240.4, 243.6)	251.4 (245.4, 257.3)	0.003
Reaction time variability, SD (mean, SD)	46.3 (23.1)	45.8 (45.1, 46.5)	51.8 (49.5, 54.2)	<0.05

Note. The N and % refer to the available N and valid % (percentage of the available data) before multiple imputation of missing data prior to the Cox regression models.

In Table 2 we depict baseline characteristics of study members according to reaction time. Taken together, shorter reaction time was associated with more favourable levels of some baseline characteristics (e.g. occupational grade and poverty/income ratio) but not others (e.g. smoking and alcohol drinking). Slower participants tended to have more variable reaction time scores, as indicated by the strong positive correlation between both measures (r = 0.64, p<0.001).

**Table 2:** Baseline characteristics of the Analytic Sample According to Mean Reaction Time.

	Slow	Medium	Fast^a^	P-value^b^
	(N = 1712)	(N = 1711)	(N = 1711)	
	N (valid %)	N (valid %)	N (valid %)	
Male	581 (33.9)	757 (44.2)	1004 (58.7)	<0.001
Ethnic minority	1252 (73.1)	1056 (61.7)	1010 (59.0)	<0.001
School grade 10 not completed	510 (30.1)	302 (17.7)	241 (14.2)	<0.001
Low occupational class	585 (36.2)	578 (35.8)	401 (25.4)	<0.001
At or above poverty threshold	485 (28.3)	360 (21.0)	275 (16.1)	0.02
Current regular smoker	482 (34.7)	482 (36.1)	484 (36.4)	0.03
> = 6 alcohol drinks per day	18 (1.4)	32 (2.2)	38 (2.6)	0.03
Physically inactive	427 (38.4)	293 (26.8)	219 (19.0)	0.001
Overweight or obese	1061 (62.1)	970 (56.7)	969 (56.7)	0.001
C-reactive protein >3 mg/dL	17 (1.1)	15 (0.9)	12 (0.7)	0.33

	Mean (SD)	Mean (SD)	Mean (SD)	P
Age in years (mean, SD)	37.6 (11.2)	36.6 (10.8)	36.0 (10.9)	<0.001
Poverty/income ratio (mean, SD)	2.0 (1.6)	2.5 (1.8)	2.8 (1.8)	<0.001
Saturated fat, g/day (mean, SD)	26.1 (17.9)	29.2 (18.0)	31.4 (19.6)	<0.001
Systolic blood pressure, mmHg (mean, SD)	118.4 (15.2)	118.0 (14.4)	119.6 (14.2)	0.02
Diastolic blood pressure, mmHg (mean, SD)	73.9 (10.6)	74.1 (10.6)	74.8 (10.6)	0.01
Cholesterol, mg/dL (mean, SD)	5.1 (1.1)	5.1 (1.1)	5.1 (1.1)	0.72

*Note*s.

a Fast/medium/slow groups derived from tertiles of simple reaction time.

b P value for linear trend.

Results from the Cox Regression analyses for the associations between reaction time and mortality are shown in Table 3. After adjusting for age, sex and ethnic minority status, being 1 SD slower on reaction time was associated with a 25% increase in all-cause mortality risk (HR = 1.25, 95% CI 1.12, 1.39). A significant association was observed for CVD mortality (HR = 1.36, 95% CI 1.17, 1.58) but not cancer mortality for which there was no significant relation with reaction time (HR = 0.85, 95% CI 0.54, 1.34). In fully adjusted models which also adjusted for educational attainment, occupational grade, poverty/income ratio, health behaviors and CVD risk factors, the association was attenuated but remained statistically significant for all-cause mortality (HR = 1.15, 95% CI 1.02,1.29; 37% attenuation), and CVD mortality (HR = 1.22, 95% CI 1.15,1.29; 36% attenuation).

**Table 3:** Hazard Ratio (95% Confidence Intervals) for the Relation of Reaction Time Measures with Mortality.

	1 SD slower reaction time					1 SD more variable reaction time				
	All-cause mortality	a	CVD mortality	a	Cancer mortality	All-cause mortality	a	CVD mortality	a	Cancer mortality
Model 1. Adjusted for age, sex and ethnic minority status	1.25 (1.12,1.39)		1.36 (1.17,1.58)		0.85 (0.54,1.34)	1.36 (1.19,1.55)		1.50 (1.33,1.70)		0.99 (0.72,1.34)
Model 2 (Model 1+educational attainment)	1.17 (1.04,1.31)	31%	1.25 (1.07,1.47)	27%	0.81 (0.50,1.32)	1.29 (1.13,1.48)	17%	1.42 (1.27,1.59)	14%	0.96 (0.70,1.32)
Model 3 (Model 1+occupational grade, poverty/income	1.17 (1.05,1.30)	29%	1.26 (1.07,1.48)	25%	0.81 (0.49,1.35)	1.29 (1.13,1.47)	17%	1.39 (1.22,1.58)	19%	0.97 (0.69,1.36)
Model 4 (Model 1+smoking, alcohol, diet and activity	1.23 (1.09,1.37)	8%	1.32 (1.11,1.58)	9%	0.85 (0.52,1.39)	1.33 (1.16,1.51)	8%	1.47 (1.27,1.69)	6%	0.99 (0.72,1.36)
Model 5 (Model 1+BMI, SBP, DBP, cholesterol, CRP	1.22 (1.10,1.35)	10%	1.32 (1.14,1.53)	10%	0.83 (0.52,1.32)	1.34 (1.18,1.52)	5%	1.51 (1.33,1.72)	−1%	0.97 (0.72,1.31)
Model 6 (Model 1+all variables	1.15 (1.02,1.29)	37%	1.22 (1.15,1.29)	36%	0.81 (0.48,1.37)	1.25 (1.09,1.44)	27%	1.35 (1.16,1.58)	25%	0.97 (0.69,1.34)

*Note*. CVD = cardiovascular disease, HR = hazard ratio, CI = confidence interval, BMI = Body Mass Index, SES = socio-economic status, SBP = Systolic Blood Pressure, DBP = Diastolic Blood Pressure, CRP = C-reactive Protein.

a The % shown are the percent attenuation of to the beta coefficient (logit), not the hazard ratio, comparing each model to model 1 (all-cause and CVD mortality).

Having 1 SD more variable reaction time was also associated with all-cause mortality, increasing risk by 36% (HR = 1.36, 95% CI 1.19, 1.55), adjusting for age, sex and ethnic minority status. The association was somewhat stronger for CVD mortality (HR = 1.50, 95% CI 1.33, 1.70). Again, there was no significant relationship between this component of reaction time and cancer mortality (HR = 0.99, 95% CI 0.72, 1.34). In fully adjusted models, the association was attenuated but remained significant for all-cause (HR = 1.25, 95% CI 1.09, 1.44; 27% attenuation) and CVD (HR = 1.35, 95% CI 1.16, 1.58; 25% attenuation) mortality. Associations were only slightly attenuated in additional models for reaction time variability in which simple reaction time was controlled for, allowing for the fact that participants with slower reaction times tended to have more variable reaction times (Table S2). This suggests that the association between reaction time variability and mortality is not simply accounted for by the tendency of those with more variable to have slower reaction times. Reaction time mean was not significantly associated with mortality after adjustment for variability (Table S2) suggesting that reaction time variability was driving the association.

The effect sizes were generally similar when analyses were performed on a nested sample of participants with complete data (Table S1), adjusting for age, sex and ethnic minority status. One exception was all-cause mortality and reaction time mean, which was markedly stronger among complete case data (HR = 1.68, 95% CI 1.28, 2.20). The general pattern of results and conclusions drawn were largely unaffected. Repeating results after excluding participants who died within five years of cognitive assessment weakened the associations (HR for reaction time mean = 1.14, 95% CI 1.03, 1.26; HR for reaction time variability = 1.19, 95% CI 1.19, 1.07, 1.31), but had little influence the overall pattern of findings, mitigating concerns about reverse causation. Results were similar across age groups (Table 4).

**Table 4:** Hazard Ratio (95% Confidence Intervals) for the Relation of Reaction Time Measures with Mortality in Three Age Groups.

			1 SD slower reaction time			1 SD more variable reaction time		
Adjusted for age, sex and ethnic minority status	N	Number of deaths	All-cause mortality	CVD mortality	Cancer mortality	All-cause mortality	CVD mortality	Cancer mortality
Model 1. Age 20 to 30	1795	51	1.25	1.30	0.58	1.26	1.32	1.22
			(1.12,1.39)	(0.90,1.87)	(0.30,1.14)	(0.90, 1.75)	(0.94,1.85)	(0.97,1.53)
Model 2. Age 31 to 42	1776	103	1.24	1.51	1.02	1.14	1.40	0.85
			(1.07,1.44)	(1.26,1.80)	(0.64,1.63)	(0.95, 1.38)	(0.98,2.00)	(0.62,1.16)
Model 3. Age 43 to 59	1563	223	1.26	1.34	0.83	1.43	1.53	0.98
			(1.11,1.44)	(1.10,1.64)	(0.46,1.48)	(1.20, 1.70)	(1.32,1.77)	(0.68,1.43)

*Note*. CVD = cardiovascular disease, HR = hazard ratio, CI = confidence interval, BMI = Body Mass Index, SES = socio-economic status, SBP = Systolic Blood Pressure, DBP = Diastolic Blood Pressure, CRP = C-reactive Protein.

## Discussion

In a representative sample of adults, slower and more variable performance on a simple reaction time task was associated with increased rates of both all-cause and cardiovascular disease mortality over a follow-up period of approximately 15 years. The association between reaction time variability and mortality remained after adjustment for reaction time mean, and was therefore not accounted for by the tendency for people with more variable reaction times to have slower responses. No association was observed for cancer mortality, although fewer deaths were available for this outcome. Socio-economic status, health behaviors and established CVD risk factors partly but not fully explained these associations.

The strengths of the study include the range of covariates considered, some of which occur between reaction time and survival. For this reason, we calculated their contribution to the attenuation of the association separately to avoid over-adjustment. No variables attenuated the associations fully, suggesting that the association between simple reaction time and mortality is independent of socio-demographic, socio-economic, health behaviors and CVD risk factors. The pattern of results was similar to those found in the Health and Lifestyle Survey (HALS) [4], [5], the Twenty-07 Study [2], and also to the Baltimore Longitudinal Study of Aging [1]. For example, in the Twenty-07 study, reaction time mean and variability both predicted mortality, consistent with our findings [2]. These studies also considered choice reaction time. Simple reaction time scores were averaged over 50 trials and thus the reliability of scores in this study are greater than those of the other studies which averaged over 20 trials.

To our knowledge, this is the first study to estimate the association between simple reaction time (mean and variability) and mortality in a representative sample of the US community-dwelling population. Our study replicates findings in the UK population [2], [3]. The associations we found for simple reaction time converge with those found in other studies. Data were not available on choice reaction time, but it is likely that simple reaction time mean and variability are less susceptible to confounding than choice reaction time. Choice reaction time involves choosing between stimuli and responding with several response options. This involves more complex cognitive processes and decision-making than simple reaction time. Study limitations include the lack of statistical power available to consider other specific causes of death, particularly cancer. Since cancer is not a single disease entity, site-specific cancers may have different associations with reaction time [42]. Analysis of site-specific cancer risk was not possible given the relatively small number of cancer deaths. Reaction time was only measured once at baseline, and so we were not able to adjust for changes in the exposure over follow-up or consider time-varying confounders. Reaction time scores are stable in the short [32] to medium term [43], but show age-related decline [44]. Although we proposed several variables as possible mediators and evaluated by how much they attenuated associations between reaction time and mortality, mediation is not straightforwardly assessed in cross-sectional data [45] and so longitudinal repeated measures of these covariates would be informative. Another limitation is that age-related cognitive decline may have occurred prior to baseline, particularly for older adults in the sample [46]. In descriptive analyses, the often weak and inconsistent relation between reaction time and covariates is likely to account for why these variables explained relatively little of the association with mortality. There may be further explanatory variables or effect modifiers that were not included in our models [43]. However, the fact that results were very similar when re-run on participants with complete data provides support for the view that results were not influenced by missing data patterns. The two exceptions in complete case analysis, a stronger association between reaction time mean in relation to all-cause mortality and between reaction time variability in relation to cancer mortality, could have been biased by non-ignorable missing data patterns. The fact that the sample were relatively young is both a strength and a limitation – reaction time could be measured before the onset of disease and death, but relatively few deaths occurred over follow-up because the sample were young. Finally, there are likely several confounding factors that were not considered in our analysis. Residual confounding could have introduced bias.

Mechanisms underlying the association between slower and more variable reaction times and mortality risk are not known. One hypothesis concerns ‘system integrity’, which suggests that since bodily systems deteriorate with age, slower and more variable reaction times reflect a central nervous system that is deteriorating in parallel with other bodily systems [2], [47]. Given the correlated heterogeneity in the aging of these systems, slower and more variable reaction times in adulthood might indicate poor physiological functioning across several bodily systems, any of which might increase risk of death in turn [48]. Simple reaction time, being less proximal to cognitive abilities than choice reaction time, might be an indicator of system integrity. It is likely however, to be one of several possible markers, and depends on whether simple reaction time actually measures functioning in one, or several systems. This question can be addressed if researchers consider if, how and why reaction time reflects functioning in other systems both cross-sectionally and longitudinally.

Our results demonstrate that slower and more variable reaction times are predictors of mortality risk in a representative population sample. Priorities for future research should include identifying the mechanisms underlying these associations. Since reaction time can be measured at low cost relatively quickly [32], it should be measured routinely in epidemiological studies.

## Supporting Information

Table S1 **Comparison of Hazard Ratios (95% Confidence Intervals) in the Main Analysis (Multiple Imputation) with Complete Cases.**
(DOCX)

Table S2 **Hazard Ratios (95% Confidence Intervals) for Reaction Time Mean and Variability Mutually Adjusted.**
(DOCX)
