# Supplementary material for: Reaction Time and Mortality from the Major Causes of Death: The NHANES-III Study
Source: PLoS One. 2014 Jan 29;9(1):e82959. doi: 10.1371/journal.pone.0082959 (PMC3906008; doi:10.1371/journal.pone.0082959)
Supplement: Table S1 — Comparison of Hazard Ratios (95% Confidence Intervals) in the Main Analysis (Multiple Imputation) with Complete Cases. (DOCX) [file pone.0082959.s001.docx]

**Supplementary Table 1. Comparison of Hazard Ratios (95% Confidence Intervals) in the Main Analysis (Multiple Imputation) with Complete Cases**

|  | 1 SD slower reaction time | | | 1 SD more variable reaction time | | |
| --- | --- | --- | --- | --- | --- | --- |
|  | All-cause mortality | CVD mortality | Cancer mortality | All-cause mortality | CVD mortality | Cancer mortality |
| Multiple imputation (main analysis) | 1.25 (1.12,1.39) | 1.36 (1.17,1.58) | 0.85 (0.54,1.34) | 1.36 (1.19,1.55) | 1.50 (1.33,1.70) | 0.99 (0.72,1.34) |
| Complete case analysis | 1.36 (1.13,1.63) | 1.68 (1.28,2.20) | 0.74 (0.42,1.29) | 1.29 (1.02,1.62) | 1.59 (1.22,2.08) | 1.20 (0.81,1.77) |

Note. Adjusted for age, sex and ethnic minority status.
