# Supplementary material for: Reaction Time and Mortality from the Major Causes of Death: The NHANES-III Study
Source: PLoS One. 2014 Jan 29;9(1):e82959. doi: 10.1371/journal.pone.0082959 (PMC3906008; doi:10.1371/journal.pone.0082959)
Supplement: Table S2 — Hazard Ratios (95% Confidence Intervals) for Reaction Time Mean and Variability Mutually Adjusted. (DOCX) [file pone.0082959.s002.docx]

**Supplementary Table 2. Hazard Ratios (95% Confidence Intervals) for Reaction Time Mean and Variability Mutually Adjusted**

|  | 1 SD more variable reaction time  + adjustment for reaction time mean | | | 1 SD slower reaction time + adjustment for reaction time variability | | |
| --- | --- | --- | --- | --- | --- | --- |
|  | All-cause mortality | CVD mortality | Cancer mortality | All-cause mortality | CVD mortality | Cancer mortality |
| Model 1. Adjusted for age, sex and ethnic minority status | 1.33 (1.15,1.53) | 1.43 (1.14,1.78) | 1.12 (0.86,1.44) | 1.05 (0.91,1.20) | 1.20 (0.93,1.54) | 0.78 (0.49,1.24) |
| Model 2 (Model 1 + educational attainment) | 1.30 (1.13,1.50) | 1.40 (1.13,1.74) | 1.11 (0.86,1.43) | 0.99 (0.86,1.14) | 1.11 (0.84,1.46) | 0.75 (0.46,1.22) |
| Model 3 (Model 1 + occupational grade, poverty/income) | 1.29 (1.11,1.49) | 1.35 (1.07,1.70) | 1.11 (0.85,1.46) | 1.00 (0.88,1.15) | 1.15 (0.89,1.48) | 0.75 (0.46,1.23) |
| Model 4 (Model 1 + smoking, alcohol, diet and activity) | 1.29 (1.12,1.49) | 1.40 (1.11,1.75) | 1.11 (0.86,1.44) | 1.05 (0.91,1.21) | 1.20 (0.93,1.54) | 0.87 (0.53,1.43) |
| Model 5 (Model 1 + BMI, SBP, DBP, cholesterol, CRP) | 1.33 (1.16,1.53) | 1.48 (1.18,1.85) | 1.11 (0.86,1.42) | 1.02 (0.89,1.15) | 1.16 (0.92,1.47) | 0.77 (0.48,1.23) |
| Model 6 (Model 1 + all variables) | 1.25 (1.25,1.26) | 1.34 (1.06,1.69) | 1.12 (0.85,1.46) | 0.99 (0.87, 1.13) | 1.13 0.89,1.44) | 0.74 (0.44,1.25) |
